# “Unconscious or for Other Reasons Incapable of Resisting”: (Un)Protected Sexual Assault Under Norwegian Criminal Law

**DOI:** 10.1177/10778012231181048

**Published:** 2023-06-15

**Authors:** Anette Bringedal Houge, Solveig Laugerud

**Affiliations:** 1 Department for Interdisciplinary Health Sciences, Institute of Health and Society, Faculty of Medicine, 6305 University of Oslo, Oslo, Norway; 2 Department of Criminology and Sociology of Law, Faculty of Law, 6305 University of Oslo, Oslo, Norway

**Keywords:** incapacitation, sexual assault, legal veridiction, rape legislation, Norway

## Abstract

According to Norwegian legislation, “sexual activity with a person who is unconscious or for other reasons incapable of resisting the act” constitutes sexual assault. Our task in this article is to identify the kind of sexual harms that are (un)protected by this paragraph and to discuss the boundaries of rape that are set by legal practice. We do so through a systematic analysis of all verdicts on incapacity and sexual assault at appellate court levels through 2019 and 2020. The analysis strengthens our concern for victims’ right to equality before the law and for the quality of courts’ legal veridiction and interpretation of both law and sexual assault.

## Introduction

In September 2020, Laugerud was observing the proceedings of a rape case in an appellate court in Norway (LB-2019-96629). As often is the case in rape case proceedings, the charges pertained to Section 291b of the Norwegian penal code, concerning sexual activity “with a person who is unconscious or for other reasons incapable of resisting the act” (The Penal Code, 2005, §291b). The defendant had been found guilty in the district court. In the appellate court, the deciding majority in the panel of judges acknowledged that the complainant was “obviously overly intoxicated” and had “a total lack of situational awareness” at the time of the alleged rape. Yet, they found “no basis to conclude that the defendant considered it most likely that the complainant was unable to resist [the sexual activity].” The appeal led to acquittal.

Seemingly irreconcilable, the deliberations in this case added to our emerging list of recent 291b cases where the judgment suggests that (a) the threshold for a person to be considered “incapable of resisting the act” varies within and across appellate courts and (b) that the threshold tends to be interpreted as higher than intended by the legislators. These observations stirred our concern that the vaguely defined “incapable of resisting” requirement leads to interpretations that challenge both defendants’ and rape victims’ equality before the law. This article presents our efforts to systematically address deliberations pertaining to the incapacity to resist requirement of all Norwegian appellate courts’ 291b decisions in 2019 and 2020. As 291b cases are at the center of the ongoing Norwegian consent debate, our task is to identify the kind of sexual harms that are (un)protected by the wording “unconscious or for other reasons incapable of resisting” and to discuss the boundaries of rape that are set by the legal system according to this paragraph and its interpretation practices.

In the following, we will briefly present the Norwegian legal context, advance the theoretical steppingstones for our analysis, and describe both the data and methods of inquiry. Building on the theoretical lens offered by Latour's concept of *legal veridiction*, we conduct a thematic analysis of all 67 appellate court decisions pertaining to 291b cases in Norway through 2019 and 2020. As the analysis shows, the appellate courts’ deliberations on resistance and incapacity beyond unconsciousness revolve around the intersection of two or more of the following contested elements that qualify an act as rape under 291b: intoxication, sleep, “freeze,” and surprise. We discuss how these elements of a potential incapacity conclusion are introduced and used in verdicts. Finally, we problematize the overall emphasis on contested situational characteristics of the complainant as a primary means through which the court decides on the culpability of the defendant.

## Norwegian Legal Context

In 2019, the 24 Norwegian rape reception centers received and assisted victims 2,039 times. That same year, the police received 1,525 reports of rape (where the complainant was above the age of 14), and we have managed to identify roughly 190 completed rape cases from the district and appellate courts combined (covering the three main sexual assault paragraphs: §291 Sexual assault, §293 Aggravated sexual assault, and §294 Grossly negligent sexual assault; see The Penal Code, 2005).^1^ Although the cases behind the number of consultations at rape reception centers, the number of police reports, and the estimate of court cases are not the same, their relative sizes suggest what seems universally true (Henry & Jurek, 2020): the justice gap is very much present also in Norway. Few victims report that attrition rates are high, and convictions few (Bitsch & Klemetsen, 2017). In this article, we zoom in on appellate court cases that decide on 291b charges.^2^ 291b cases concern charges of sexual assault against “a person who is unconscious or for other reasons incapable of resisting the act.” While the wording is vague, the preparatory deliberations specifically point out that “unconsciousness includes sleep” and that other reasons may involve “heavy intoxication, physical disabilities, mental conditions, or the like” (Ministry of Justice and Police, 2008, p. 216). However, there is no equivalent, explicit definition or boundary drawn on what defines the act of or ability to “resist” in the context of 291b cases, neither in the preparatory deliberations nor in the legal text as such.

291 cases require *mens rea*, i.e., a person can only be convicted for a crime if they knew at the time that they were committing the crime. This is a general requirement that applies to most crimes in The Penal Code. *Mens rea* is however irrelevant to Section 294, which criminalizes rape by gross negligence. In 291 cases where the court does not consider *mens rea* to be proven, Section 294 might apply. Additionally, to be convicted of rape, it must “be proven beyond any reasonable doubt” that the accused committed the rape. The standard of proof in criminal cases is often metaphorically presented as a probability close to 100%. This means that any reasonable doubt must benefit the accused. To be found liable to pay compensation requires a lower standard of proof. The courts make decisions regarding compensation based upon a preponderance of evidence, a standard of proof that is lower than criminal cases but higher than regular tort cases that require a probability above 50%. This means that verdicts in rape cases include two legal decisions in which an acquittal in the criminal case can be followed by a ruling to pay damages in the compensation case. A panel of judges, which consists of two legal judges and five lay judges, make the decisions together for both the criminal and civil cases. A conviction in the criminal case requires five votes and in the compensation case a simple majority vote. Every decision needs to be explained in the written verdict, and disagreements are accounted for in dissenting opinions in the verdict.

Importantly, 291b cases are at the center of the ongoing Norwegian consent debate, concerning the prospective inclusion in rape legislation of wording that explicitly “reflects that sexual intercourse without consent is prohibited and defined as rape” as formulated by the current minority government's cooperation platform (Regjeringen, 2021, p. 65). Proponents emphasize both the normative effects of legislation and hold that without such consent-based legislation, the law only recognizes rape if the victim slept or was unconscious, threatened, or forced through violence —excluding many rape victims from legal protection and recognition through criminal justice (see, e.g., Amnesty, 2021). Skeptics argue that the explicit inclusion of consent will increase pressures on victims to signal that they resist the act to prove that they did not consent and, further, that it will not address the key challenge behind high attrition rates: the question of proof. Legal scholars, too, emphasize how explicit (rather than implicit) inclusion of consent in the rape provision will do little to solve the difficult challenge of evidence in most rape cases (Jacobsen & Skilbrei, 2020). Consent-based rape legislation will still be characterized by the defendant's word against those of the complainant (see, e.g., Ministry of Justice and Police, 1997, 2008).

According to the Criminal Procedure Act (1981), §305, all evidence—from witnesses, documents, or evidence of objects—must be presented orally during the main hearing. The main hearing is, thus, the knowledge basis upon which the judges shall make their decision. The rules of evidence permit the parties to present any kind of evidence, except sexual history evidence and other forms of evidence that target a witness’ character or credibility in general (§134). It is against this background that we provide empirically founded commentary on the current understanding, interpretation, and application of the incapacitation requirement as it is worded in the present legislation at the level of Norwegian appellate courts.

## Theoretical Context

Two tracks of research on the legal processing of rape are of particular relevance to this study: research that focus on rape myths/rape culture on the one hand and the role of expert evidence on the other. Research into the former has examined the existence, role, and persistence of rape myths in police investigations (e.g., Garza & Franklin, 2021; Sleath & Bull, 2017), prosecutorial decision-making (Hansen et al., 2019), and court processes and convictions (Duncanson & Henderson, 2014; Temkin et al., 2018). The term “rape myths” refer to “descriptive or prescriptive beliefs about sexual aggression (i.e., about its scope, causes, context, and consequences) that serve to deny, downplay, or justify sexually aggressive behavior” (Gerger et al., 2007, p. 423). Typically, rape myths fall into one or several of four categories. Either they blame the victim and claim that the alleged victim has somehow asked for, tempted, deserved, or invited sexual assault through the way they dress, drink, walk, or talk. Or they doubt the allegations, for example, through claims that a real victim of a real rape screams, fights, gets injured, and reacts in certain and ideal ways in the aftermath: she reports the rape immediately, is broken and traumatized, but is still consistent and clear in her recollection. Moreover, it is a common rape myth that allegations tend to be false because women claim rape when they want revenge or regret having had sex. A third category of rape myths makes excuses for the perpetrator, i.e., that once aroused, their sexuality is beyond control. The fourth rape myth category refers to assumptions about where and between whom rape happens and not—such as the claim that “real rape” is committed by a stranger by means of violence (see Bohner et al., 2009; Burrowes, 2013).

Along the second track, the production and use of, trust in, and impact of rape kits (Tiry et al., 2020) have been of particular academic interest, as has forensic and technical evidence—from DNA evidence (Forr et al., 2018) and so-called date-rape-drugs (Jenkins & Schuller, 2007) to medical exam results and proof of physical injuries (Alderden et al., 2021; Rees, 2012). The role of medical and forensic expert evidence has been the object of critical academic scrutiny at the same levels of legal processing as has rape myths (Johnson et al., 2012). Overall, the entry of forensic evidence into court proceedings and the technological developments that increase their accuracy have led to a shift in legal arguments in Norwegian rape cases, from proving or disproving sexual contact to disputing whether the forensically established sexual encounter was consensual (Dahl & Lomell, 2013). Thus, despite proliferating forensic technologies, rape and sexual violence are still particularly challenging crimes to prove, often at its core dealing with questions of credibility and reliability, of words against words, rather than proving facts through physical or expert evidence. Particularly relevant to our study, research suggests that rape myths and forensics overlap in their impacts. Hansen et al. (2019, p. 59) found that “no [voluntary] victim intoxication during the assault and more physical injuries (…) increase the likelihood of case continuing for prosecution.” Similarly, Bitsch and Klemetsen (2017, p. 177) show that victims’ use of alcohol or drugs at the time of assault has “been associated with lenient sentencing practices and victim blaming.” These tracks of research on the legal processing of rape are complemented in important ways by “a large body of research on alcohol-involved sexual assault” outside of the legal context (Lorenz & Ullman, 2016, p. 90). Such research highlights the high prevalence of victim (and perpetrator) intoxication in reported rapes, as well as the complexity, ambiguity, and chaos that may characterize sexual interactions and questions of agency and culpability when alcohol is involved (Stefansen et al., 2021; Tutenges et al., 2018). This adds importance to the study of how intoxication and incapacitation are understood in court cases. We have yet to see, however, systematic research that addresses how the incapacitation requirement specifically plays out in judges’ deliberations.

We address this knowledge gap in the following analysis of judges’ interpretation of incapacitation as set out in The Penal Code Section 291b and what their verdicts establish as evidence for or against such incapacitation. Drawing on Latour (2013), we find *legal veridiction* to provide a useful framing to address and assess these deliberations. According to Latour (2013), legal veridiction refers to the process of distinguishing truth from falsity according to legal logics. To see law, and specifically the deliberations we address as (expressions of) a regime of veridiction, means that we address how law inquire into truth about contested matters according to its own distinct legal parameters—through the storied representations of witnesses, be they expert or lay witnesses’ observations. In doing so, we acknowledge how legal practice is self-referential, building on legal logic that brings into court representations of the social and physical world through statements about what happened that follow parameters set by law. Legal truths do not rely on proofs, but on legal means that follow preset rules that aim at closing a case (Latour, 2010, 2013). Following Latour (2013), legal veridiction is, thus, distinct from scientific veridiction. In cases where scientific evidence is used, the judges ensure that the expert is not usurping the role of the judge. This entails “a process of taking into account but not necessarily deferring to scientific knowledge, nor to claims of scientific objectivity and truth” (Seear & Fraser, 2016, p. 17). Legal veridiction implies that the acts considered and the truths constructed about them in court are framed, altered, and retold by law—to produce a juridically acceptable narrative about what happened, a telling that caters to the qualifying sections and phrases that constitute the social world as legally relevant and acts as crimes or not so proven (see also Houge, 2019). In 291b cases, what makes legal sense and qualifies as “incapable of resisting” is left to the court actors’ interpretation. In this vein, our epistemological approach is constructionist: “we seek to [address and] theorize the sociocultural contexts, and structural conditions, that enable” the judges’ legal meaning-making (see Braun & Clarke, 2006, p. 85). The concept of veridiction allows us to assess how the stories that judgments produce on guilt or acquittals, participation, awareness, and in/capacity reason about the social world beyond law and how they make legal sense of witness evidence. Put simply, we examine the judges’ understanding of the boundaries that negate and establish an act as rape or not according to the legal parameters set by 291b. Thus, in the analysis below, we address the basis on which judges render incapacitated rape im/probable, seeking to understand practices of inference and sensemaking from this recognition of their deliberations as a process of legal veridiction.

## Material and Methods

The article springs out of troubling observations in court that inspired us to conduct a systematic, qualitative analysis of how the incapacitation requirement of the Norwegian rape jurisdiction is engaged and understood in appellate courts.

### Data and Timeframe

We limited our analysis to verdicts in appellate courts. All decisions at this level are registered in a database operated by LovData that the University of Oslo has access to. The cases reflect the totality of appellate court decisions on 291b cases over a period of 2 years—from January 2019 to December 2020. 2019 was the first full calendar year where all appellate court decisions provided a written justification after a discontinuation of the jury system (Kolsrud, 2017). 2019 was also the last “normal” year before the COVID-19 pandemic and associated pandemic control measures altered the everyday life in Norway and the expected speed with which courts at all levels proceeded with their caseloads. 2020 is the last year where we have access to the totality of cases, considering the timeframe within which we collected data for this analysis (December 2020–October 2021).

### Case Inclusion and Exclusion

The article is based on analysis of all the LovData-registered verdicts in Norwegian appellate courts that included rape charges under Section 291b of the Norwegian penal code. In the LovData database, our free text search included the following truncated terms: “unconscious*”, “incapa*”, and/or “resist*” (translated for the present paper), while we also limited the search to criminal cases, appellate courts, and the given timeframe and restricted our extraction to the proper section of the penal code (LOV-2005-05-20-28-§291). From the outset in December 2020, we tested different variables and search criteria and ended up with these phrases to err on the safe side. That is, we preferred manually excluding surplus hits of cases without relevance according to the exclusion criteria set out below overrisking a more specified search that did not include all relevant cases.

We manually exempted verdicts from analysis for several reasons: a few of the hits were Supreme Court or district court decisions; some were registered or counted twice in the search result. Most of the cases we removed concerned either minors, incestuous relations, and/or sexual assault that formed part of a regime of domestic terror—that is, cases where 291b was either not applied or subsumed under charges that prioritized the provisions under 291a: sexual activity obtained through violence or threatening conduct. Once every other month in the project period, we conducted a control search in LovData where we cross-checked all cases with the result from the previous month. We did this to make sure we included cases that were delayed in their registration in LovData. From the first search (December 2020) to the last control search conducted on September 30, 2021, the case load increased by three verdicts. In total, a data set consisting of **67 appellate court judgments** forms the basis of this analysis. We have taken measures to secure that our analysis includes *all* 291b cases in 2019 and 2020 that pass our inclusion criteria. There are still potential sources of error. First among these is that the registration of some cases may be further delayed at appellate court levels; another is that there are registration errors or typos in verdicts that make verdicts escape our search variables. We believe such errors, if present, amount to only a few cases, after various test runs and alternative searches where we have cross-checked results.

**Chart 1.**
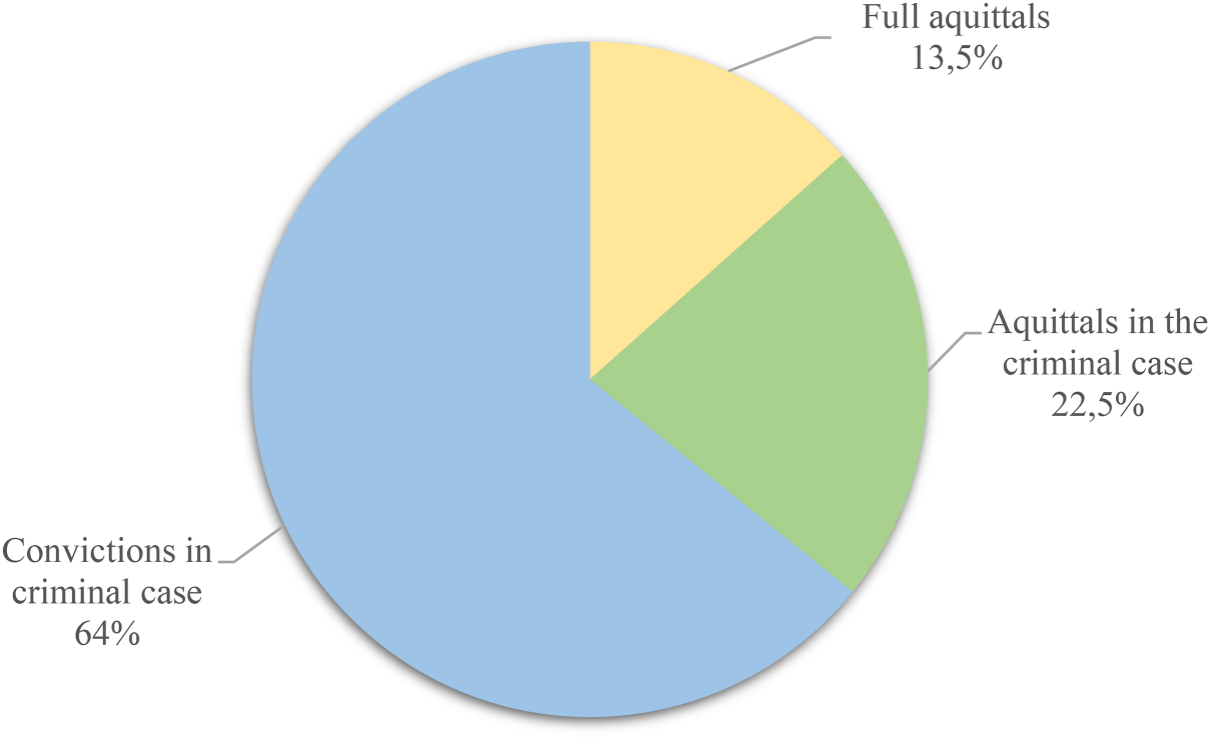
Verdicts in appellate courts.

While our analysis is qualitative, emphasizing meaning over numbers, the following simple quantifiables provide wrapping of the empirical material we have assessed: Of the 67 appellate court judgments, nine (13,5%) were full acquittals (criminal case *and* compensation), and 15 (22,5%) were acquittals in the criminal case, as displayed in Chart 1 below.

**Chart 2.**
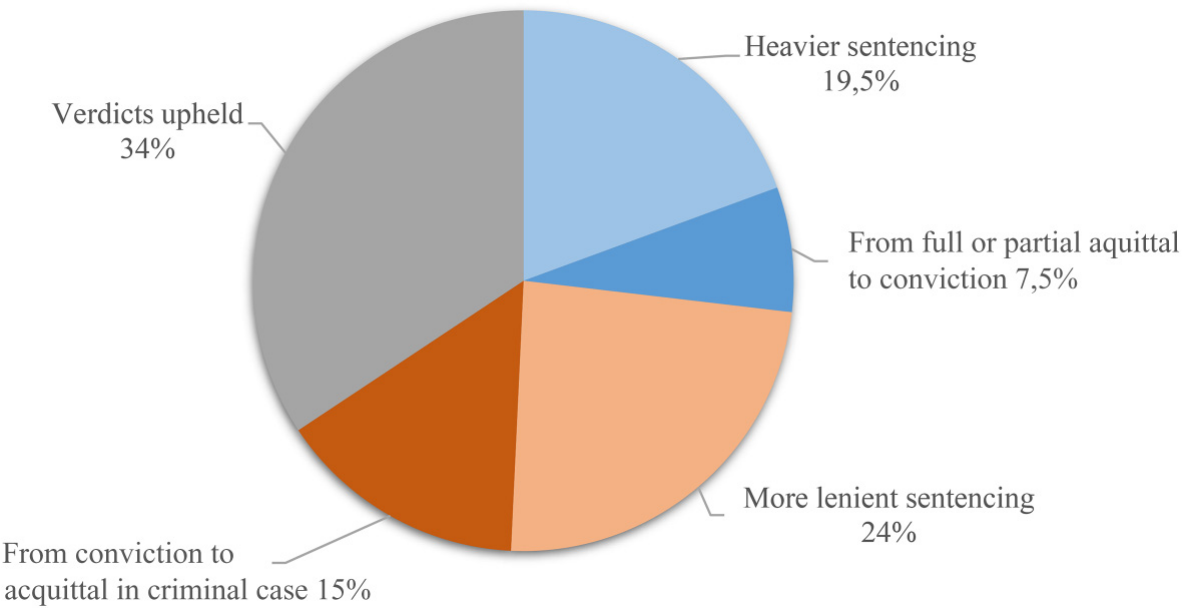
Outcome in appellate relative to district courts.

Thirty-five cases (52%) were dissent-based, out of which 10 were acquittals. As illustrated in Chart 2, 18 cases (27%) met a heavier conviction in appellate courts than at district courts, out of which five went from full or partial acquittals to conviction. In 26 cases (39%), the reverse was true: the appeal led to lighter sentences—10 of which went from conviction to acquittals. Twenty-three verdicts (34%) were sustained from district to appellate court.

The 67 cases involved 72 defendants, 33 of which were born in the 1990s and 21 of which were born in the 1980s. The complainants’ ages are not consistently accounted for in the verdicts. Twenty-three of the cases concerned charges of events that took place in 2017, 17 cases referred to acts in 2018, 11 to 2016, and 10 to 2019. In all but one case the defendants were male. In the one case where the defendant was a woman, the complainant was as well. In five cases, the complainants were men. While in 12 cases we could understand from the judgment that the defendant was a non-Norwegian citizen, there was no explicit discussion or comment on issues to do with citizenship, culture, ethnicity, or race in the judgments (see, however, Bitsch & Klemetsen, 2017).

### Analytic Process

We conducted a thematic analysis to identify what kind of sexual harms are (un)protected by the 291b paragraph and to discuss the boundaries of rape that are set by the Norwegian law and appellate courts’ legal practice. In simple terms, “thematic analysis is a method for identifying, analysing and reporting [themes] within data” (Braun & Clarke, 2006, p. 79). Building on Braun and Clarke (2019, p. 594), we understand themes as “creative and interpretive stories about the data” that articulate patterns of shared meaning across the data items or cases and that researchers produce “at the intersection of [our] theoretical assumptions,” the data themselves, and our coding practice and process. Translated to our study, we used thematic analysis to study the contextually specific social—and for our purposes, legal—construction of meaning and representation of rape, resistance, and incapacity in Norwegian appellate courts.

We did not engage in this effort in a theoretical or epistemological vacuum. It follows from our theoretical framework that our thematic analysis is at what Braun and Clarke (2006, p. 84) define as at “a latent or interpretative level.” We go beyond the semantic content of the data and seek to identify “the *underlying* ideas, assumptions, and conceptualizations” (Braun & Clarke, 2006, p. 84) of rape, in/capacity, and resistance that underpin the boundaries set by legal interpretation practices—that is, the legal veridiction processes that inform the judges’ deliberations and courts’ decisions. Moreover, we were familiar with both the research field and the political debate that revolve around sexual assault and its political and legal responses. Not least, as researchers, we have read hundreds—if not thousands—of judgments pertaining to charges of rape and/or sexual assault, and, as evident from the introduction, we have observed in numerous court cases. We still consider our thematic analysis as primarily inductive: The themes we developed through the coding process were strongly linked to and based on the data set we worked with, rather than predefined by any “preexisting coding frame” or the literature we refer to (Braun & Clarke, 2006, p. 83). We dived into the data set individually at first—reading and rereading judgments, manually extracting sections of the verdicts that provided backdrop for or formed the various arguments for or against incapacitation, ability to resist, and arguments referring or alluding to defendant and complainant responsibilities and culpabilities. In the next round, we summarized each case and added codes to the extracts—keywords that captured the main arguments of the coded text—and commented on and discussed between us aspects of the extract that stood out as particularly relevant to our analytical “quest.” As Braun and Clarke (2021, p. 331) emphasize, this analytical process was recursive. We compared and discussed our extracts, codes, notes, and case summaries, reread sections, moved back and forth in the material, and consulted with literature and theory along the way, as we combined codes and constructed themes that storied, or captured, the verdicts’ constructions of resistance and in/capacity across the data set. We found that whether verdicts ended in acquittals or convictions, deliberations revolved around specific themes relating to four incapacity and resistance qualifiers: intoxication, sleep, freeze, and surprise. Interestingly, “unconscious,” the single explicit qualifier in Section 291b of the penal code was used *only* to qualify sleep or intoxication as incapacitation, and not independently. Importantly, the qualifiers that signify and give our themes their name are not mutually exclusive. An overwhelming majority of verdicts concern intoxication (*n*  =  56) or sleep (*n*  =  53), most often in combination (*n*  =  45), and sometimes with deliberations concerning other qualifiers, that is, surprise or freeze reactions that hindered verbal and/or physical resistance.

In the analysis below, a selection of cases illustrates our findings. We identified extracts that are particularly illuminating for what we see as typical deliberations, rather than sensational ones, to shed light on recurring conceptions of incapacitation, or variations of similar deliberations. However, while we address some characteristics in deliberations that are valid across cases within a given category of incapacitation qualifiers, there is no such thing as a representative case or verdict in this material. Reading these verdicts, what most strikes us is the unpredictability of outcome given different sets of circumstances concerning incapacitation. In the presentation of findings, we have sought to balance what we see as typical or particularly illustrative deliberations with a rich representation of the specificity of the cases. Notably, many of the illustrative cases are cases decided with dissent—as the deliberations in such verdicts tend to be more thorough and nuanced.

### Ethical Considerations

The ethical management of research data and the privacy impact assessment are approved by the Norwegian Centre for Research Data (project numbers 546086 and 354820). In the preparation of this article, we have discussed whether to include case file numbers. Inclusion secures transparency and allows us to quote text extracts for illustrative purposes. Inclusion also makes it possible for readers to identify specific verdicts in LovData that concern serious charges and sensitive experiences for the implied parties. All verdicts are deidentified by LovData before publication, and in the rare event that the verdicts have not been fully deidentified due to random typos of the defendants’ or victims’ names, we have notified LovData who have swiftly secured proper deidentification. Important in this regard, too, our analysis is less concerned with the individuals involved, and more about the deliberations of the judges concerning the specific incapacity section of Norwegian rape legislation and its applicability in categorized cases. While judges are not deidentified in publicly available verdicts, their deliberations and the quality and foundation of their reasoning are of explicit public interest. We have, therefore, concluded that the inclusion of case file numbers and translated text extracts is not only permissible but also necessary.

## Findings

With a few exceptions, 291b cases typically concern sexual encounters between acquaintances and involve alcohol and/or sleep, and the sexual contact is not contested. None of the analyzed cases considered “unconsciousness” in isolation. Rather, unconsciousness is consistently coupled with “other reasons” that cause incapability of resisting. In the following, we analyze and provide examples of where the line of in/capacity is drawn in relation to the four main categories for incapacity to resist that the appellate courts address: intoxication, sleep, “freeze,” and surprise. The categories overlap: sleep tends to combine with alcohol, freeze with surprise.

### Intoxication: Drunken Complainants, Unreliable Victims, and Toxic Evidence

A typical case concerns sexual contact at a time when both the defendant and complainant were substantially intoxicated. Out of the 67 verdicts, 55 (82%) concerned alcohol and/or other forms of intoxication, a majority of which also involved sleep (45). All full and partial acquittals in our material concerned alcohol and/or other forms of intoxication, including all cases that went from conviction at the district court level to acquittal on appeal. In all cases, intoxication referred to alcohol, sometimes in combination with other drugs, proscribed or not. In several of these cases, the deliberations pertaining to in/capacity to resist resembled those of the case that introduced this article: the appellate court was convinced that the complainant was “overly intoxicated,” yet questioned the extent to which the complainant could not resist, as well as the extent to which the defendant knew or should have known that the complainant could not resist their acts.

The following cases demonstrate how the judges recognized the victims’ experience of what happened, but did not trust that their accounts reflected the reality of the situations described. LB-2020-41177 provides an example of such a case that ended in a full acquittal. Here, the defendant was charged with attempted rape at an afterparty in his apartment. Both he and the complainant were heavily intoxicated. According to the verdict, the complainant had no recollection of how they ended up in the apartment but remembered how the defendant physically prevented her from leaving and attempted to forcibly rape her. The judges stated that they had no doubt that the complainant testified truthfully and experienced the defendant's acts as a rape attempt. The majority concluded, however, that they could not rule out the explanation of the defendant. He, on his side, acknowledged that he was drunk and that he could not remember all the details, yet portrayed both himself and the complainant as active sexual partners. He also admitted that he “wrestled” the complainant down onto the couch when she tried to get up. According to the verdict, he consistently acknowledged that he overstepped a boundary by doing so, as she turned her head away from him, physically resisted, and verbally expressed that she did not want to give him a “blowjob.” In their deliberations, the majority still concluded that the proceedings had not proven that this constituted a rape attempt. Following the deliberations of the majority, there had not been presented evidence that “beyond any reasonable doubt, gave [the defendant] *reason to refrain from continuing sexual intercourse with* [the complainant] because of her reluctance.” Moreover, the majority held the complainant to have been more intoxicated than she believed herself—which implied that they could not rule out the chance that she had forgotten important details of the evening. Yet, as it follows from the acquittal of the defendant, she was still not deemed to have been intoxicated *enough* to be considered incapacitated as required by 291b (see also LB-2020-41177, LG-2018-190076). The case is atypical for its violence and the defendants’ admitted boundary-crossing behavior and as it does not involve consummated rape—but illustrative of how courts often find the complainant to be credible but unreliable (due to her lack of memories). It is also illustrative of the lengths the courts go to construct and give the defendant the benefit of doubt: Even when the defendant admitted that the complainant physically and verbally resisted, she was not considered to have provided the defendant with “*reason to refrain from continuing sexual intercourse*.”

LA-2019-173370 provides another example of a court case where the verdict concluded that the complainant was trustworthy, yet unreliable. The case concerns an alleged rape during a business trip, involving a 19-year-old apprentice and her senior male colleague. On their way from one bar to the next, they stopped at a table in a backyard partly out of sight from the street, where the contested sexual intercourse took place. A witness interfered and questioned what was going on because the complainant appeared “completely lifeless.” Despite the witness’ intervention, the complainant had been lying flat and unresponsive on her back on the table with her head turned away and her dress pulled up to the waist, and she had not tried to cover herself. The defendant got angry at the witness, but during their heated exchange, the complainant had still not reacted in any way. The court accepted that the complainant was intoxicated at the time of the incident and found her credible, or as the verdict put it: “The Court has no reason to believe that the aggrieved party deliberately explains herself incorrectly.” Still, the court acquitted the defendant:

When the Court still does not find it proved beyond any reasonable doubt that the incident amounted to rape as described in the indictment, it is based on an assessment of the overall evidence. The Court also finds the defendant's explanation credible, albeit somewhat more adapted to the situation than that of the complainant. [The Court] cannot rule out that the defendant's explanation is correct.

According to the court, the complainant's own explanation was central to the acquittal. She claimed to have asked the defendant repeatedly, “Don’t you have a family?” but admitted that she never physically resisted, called for help, or stated “no.” The court found that this could not give “the accused the impression that she opposed or did not want the sexual intercourse.” The court appears to have considered the complainant's ability to talk evidence that she was able to and should have directly resisted verbally and physically. In doing so, the court was ignorant of potential power imbalances in play (in terms of age and position in the company), and potential fear of what blatant rejection could lead to. Further, the court found no evidence that the complainant was, at any point, unconscious or for other reasons incapable of resisting the act, as required by 291b. She remembered what happened too well for that to be the case. To the contrary, the court concluded: “[The complainant's] own explanation of what she remembers of what happened in the garden is so detailed that it can hardly be assumed that she was incapable of resisting the act, legally speaking” (LA-2019-173370). The court appears to have equated the incapacity requirement with a state of complete unconsciousness.

The above cases illustrate how complainants’ memories constitute a tricky challenge in 291b cases involving intoxication. One illustrates how the complainant is deemed too drunk for her memories to be reliable, while the other illustrates how a too detailed memory is considered evidence that the complainant was not incapacitated. The paradox is created by the problematic qualifier that is the phrase “incapable of resisting.” When the threshold for incapacity is at the level of unconsciousness, ignorant of other factors that may inhibit the victims’ ability to resist, or influence the form of that resistance, the courts expect complainants to manifestly and explicitly, physically and verbally, object and resist sexual advances unless they are practically comatose. Such case law suggests that in the absence of explicit, fierce objections, a defendant can assume that a drunk but somewhat awake complainant wants to engage in sexual activities at any given time—with the law on their side. This is evident in cases that end with convictions, too: emphasis is on the quality and intensity of the victims’ objections, not on the defendants’ opportunistic and exploitative behavior in a “gray zone”^3^ of severe intoxication that reduces complainants’ capacity to manifestly object. It appears as if the courts, when narrowly interpreting incapacity as unconsciousness, turn their gaze toward *acts* (of resistance), rather than the complainants’ *in/capacity* to *resist*. In this process of legal veridiction, the ability to act or utter is seen *as* capacity to resist: Evidence of any mental capacity or talk is interpreted as capacity to verbally resist, and evidence of bodily movements is interpreted as capacity to physically resist. Once this threshold of capacity is established, it becomes an apparent excuse for the defendant to pursue sexual advances. This alludes to two typical rape myths: one that assumes that real victims of rape screams, fights, and gets injured and another that presents the defendant as someone who will necessarily act on his sexual impulses unless he is expressly prevented.

At times, deliberations in intoxication cases that end in convictions also illustrate that a complainant's verbal objections do not suffice as resistance. LH-2019-155441 provides such an example. The case concerns a woman who fell ill from intoxication at an afterparty, vomited at the bathroom, and remained on the bathroom floor afterward. The defendant came into the bathroom and ignored her objections to his advances. The court emphasized that a combination of intoxication and nausea made the complainant “completely unable *to get away* when the accused abused her” (emphasis added) that the complainant told the defendant that she did not want to have sex with him, and tried to push him away, “but because she was drunk and nauseous, she could not resist *more than that*.” Whereas the defendant in the former case was acquitted because the complainant had not said “no,” here, the defendant was convicted—not because the complainant explicitly said “no” but because she was unable to resist *more* and *get away* from the defendant, because of heavy intoxication. It further helped the complainant's case that the defendant denied having had sex with her altogether, despite technical evidence to the contrary. Although rare, some defendants stick to their initial denial of any sexual intercourse, despite forensic discovery of the defendants’ semen and DNA at the examination of the complainant at the rape reception center. In such cases, the defendants present various forms of secondary transfer stories that are rarely found credible by the court (see, e.g., LB-2018-169937 and LB-2018-98851).

In 12 intoxication cases, the verdicts referred explicitly either to toxicological reports or to the testimony of toxicologists as expert witnesses in the case. Toxicologists are usually asked to calculate the degree of intoxication at the time of the alleged rape and to explain the consequences of different levels of intoxication on the complainant's body and mind. We expected that unconsciousness based on intoxication would join practices in law and science to build legal truths about incapacitated rape. Usually, toxicology reports are included to confirm the complainant's explanation. In some cases, however, incompatibilities appear between the forensic account and witnesses’ accounts. Instead of creating tensions, the courts combine these differences to support specific accounts of the incidence in question. LB-2019-17336 is a case in point. Here, three men were convicted for raping a young woman. Many witnesses, including two of the defendants, described her as too drunk to stand on her feet, as lying on the floor unable to move, that she was carried to a bedroom because she could not take care of herself, and that it was impossible to communicate with her. The forensic expert, however, concluded that she (only) had 1.0–1.3 per mille alcohol concentration in her blood at the time of the offense. According to the toxicologist, an alcohol concentration at this level suggests “mild to moderately impaired consciousness,” although there can be significant individual variation. Based on these observations, the court concluded:

After an overall assessment of the evidence in the case, cf. several witness testimonies accounted for above, there is no doubt that the symptoms that the court has found proven of the complainant's state, are in accordance with the condition *significantly reduced consciousness.* (LB-2019-172336, emphasis added).

The court thus *dis*connected the expert's calculation of alcohol concentration with the category “mild to moderately impaired consciousness” on the toxicological scale of reduced consciousness, *replaced* the calculated per mille concentration with the witnesses’ descriptions of the complainant's state, and re*connected* the witness descriptions with the toxicological scale of consciousness at a different level: “significantly reduced consciousness.” This way, the court enacted the toxicological scale of reduced consciousness—that is, toxicological *lingo*—but exerted legal discretion in its interpretation of toxicological *facts*. The court accordingly relied on traditional legal evidence: witness testimonies. This practice of legal deliberation, that is, the plasticity and eclectic use of toxicological evidence, makes it possible to combine the authority of science with storied observations of what happened to produce a legal truth of rape (see also, e.g., LG-2019-75089). This way, judges also ensure legal veridiction by not having their role usurped by the scientific expert (Latour 2010; 2013).

A central characteristic of the 291b cases that involve intoxication revolves around the issue of sleep and recurring deliberations on whether the complainant was asleep, how heavy, and if they woke up during the alleged assault. The second main category that qualifies the state of a complainant for incapacity to resist, is, accordingly, sleep.

### Sleep: Sleeping Complainants and Commonsensical Evidence

In Norwegian, “sleep-rape” is a common term that refers to sexual assaults committed against sleeping—and often intoxicated—victims. These assaults tend to be party-related and are prevalent in 291b cases. Out of the 67 verdicts, 53 (79%) concerned sleep, 45 of which also included intoxication. Six of these cases ended in full acquittals, whereas 47 verdicts convicted for compensation, out of which 41 cases led to a conviction in the criminal case. In 20 of the cases, the appellate courts upheld the verdict from the district level. Eighteen cases led to a lighter sentence in appellate courts than at trial, while the reverse was true for 15 cases.

In sleep deliberations, a variety of sleep norms and incapacity interpretations come into play. In some cases, a conviction hinges on whether the complainant was deep enough asleep for it to qualify as unconsciousness. One such case concerned a party-related sexual assault, where the complainant first fell asleep on a coach, heavily intoxicated, before she was supported into a bedroom, where she slept on (LB-2019-50934). She woke up 2 hours later with the defendant's penis inside her vagina, shocked, in pain, and scared. The defendant claimed that he perceived her to be awake and that she invited the sexual contact by way of moving her body and moaning. In the verdict's deliberations, the judges pointed out that “‘unconscious’ includes sleep or intoxication, or a combination of sleep and intoxication” and that the issue to be decided was “whether the complainant was ‘unconscious’ when the defendant had sexual intercourse with her, in other words whether the defendant exploited a state of helplessness in the victim.” Notably, the court did not consider whether she was unable to resist. The judges found that the complainant was heavily asleep as the defendant entered the bedroom and further rejected the defendant's claim that her bodily reactions signified that she was awake. As the verdict stated:

Although it is not uncommon to move, moan, and even say simple words like “yes” in your sleep, in the Court's view it is completely unlikely that a person who wakes up from sexual advances will continue to keep their eyes closed until and while the sexual intercourse takes place.

Here, the court acknowledged that bodily reactions are not indicative of consciousness, but also enacted commonsensical reasoning about victims’ behavior during assault that engaged norms pertaining to sexual behavior, and alluded to rape myths of “proper” victim responses when assaulted.

Sleep norms, too, seem to inform and shape court deliberations and legal practice when courts consider whether a complainant was incapacitated. The court rarely draws on expert testimony to consider unconsciousness or incapacity in terms of sleep or dormancy. Rather, sleep is an issue subjected to the judges’ commonsensical reasoning, an important part of court deliberations as such. Both intuitive and self-evident, the rhetoric of common sense, make it powerful and difficult to challenge, even in a court setting (Cochran, 2013; Laugerud, 2020a; Moran, 2003). In the context of incapacity, norms regarding (in)appropriate sleep appears to be guiding meaning-making practices in deliberations. Sleep is perhaps not something commonly associated with norms, but what people can(not) do in their sleep, as well as when, where, and how to sleep, is still a matter of common concern (Williams, 2007). In several court deliberations, sleep norms seem to both inform and shape credibility assessments. One example of such reasoning concerns norms regarding how fast complainants are likely to fall asleep and how easy they (should) wake up again. In a dissenting decision, the decisive minority acquitted the accused based on an assumption that people do not, and accordingly should not, fall into heavy sleep too quickly:

The minority points out that the accused's intercourse with the complainant happened shortly after [the complainant had intercourse with another named man]. Although she was somewhat intoxicated, it is not clear how she could have fallen into heavy sleep this quickly so that she could not resist the intercourse. (LH-2019-136465)

While the defendant was not judged for not making sure the complainant was awake, the complainant seems to be judged on the basis of how well they abide by certain sleep norms, rather than whether (s)he was in fact incapacitated.

In LB-2018-119700, another party-related case, the court similarly made the amount of time passed from the complainant was awake until she allegedly was asleep a decisive factor. Here, the complainant and defendant were former, occasional lovers who ended up in the same bed after partying. According to the verdict, the complainant was drunk, rejected the defendant's sexual advances, but accepted that they “spooned” as they lay down. The appellate court rejected the defendant's claim that he did not penetrate the complainant with his penis, but the majority found that they could not rule out (the relevance of) his claim that he did not at all consider whether the complainant was asleep as he penetrated her, making the case primarily one about his *mens rea* (see also LB-2019-171089). Shortly before the charged acts, the verdict reads, the complainant got out of bed, into the bathroom, and vomited from intoxication. As she lie down again, they were spooning, and the majority pointed out:

[The complainant] was accordingly lying with her back against [the defendant], and he could not see her face, and therefore could not see if she was asleep. [The defendant] has explained that it was dark in the room.

By pointing out the short time span from the complainant's visit to the bathroom on the one hand and the charged acts on the other, the majority allowed the peculiar combined defense of darkness and not even considering whether the complainant was asleep possible. In doing so, the majority ignored both the reasons for the bathroom visit (to vomit because of intoxication) which suggested that she could fall fast asleep, as well as her clear rejection of his advances. In the deliberation of the verdict, the majority pointed out that he “*should* have made sure that [the complainant] was awake *and capable of resisting intercourse* prior to penetrating her vagina with his penis” (emphasis added) and that “his behavior warrants strong reproach for lack of care.” Moreover, the majority remarked that they could not rule out that the complainant woke up as the defendant penetrated her vagina with his penis and that the penetration therefore lasted very briefly. On these grounds, the appellate court reduced the sentence from district court, finding him guilty of grossly negligent rape (§ 294) rather than rape with intent. It is noteworthy how the court applied the wording of Section 291b to emphasize his lack of consideration to her *capacity to resist* prior to penetration—illustrating the threshold for legal sexual encounters at the level of resistance, not desire. The court deliberations in this case indicate a problematic demarcation for what constitutes rape and not, again illustrating how the threshold is not at consent or mutual desire, but at the qualifier “resistance,” as also accounted for above.

When and how the complainant wakes up are also crucial in some decisions in determining whether she was unconscious or for other reasons incapable of resisting the act. An unexpected and strong reaction during the alleged rape tends to be considered in favor of the complainant (see, e.g., LH2019-119406), while falling asleep too soon—as in the cases above, or with a stranger in the room (as in LG-2020-50105 below), or not waking up from advances or from being undressed (e.g., LB-2018-125155)—is considered questionable. It follows that sleep norms appear to contribute to an increased responsibilization of incapacitated people.

Moreover, sleep is in several cases constructed as the exclusively acceptable state of the complainant that allows the court to consider them “incapable of resisting.” In LG-2020-50105, for example, the court did not consider whether the complainant in any way wanted to engage sexually with the defendant, but rather focused on the extent to which her state during the assault could undoubtedly be defined as sleep. According to the verdict, the complainant was out in town and got severely intoxicated and lost, while her phone ran out of battery. A friend of the defendant, and stranger to the complainant, found her freezing, trembling, and thinly dressed, and invited her inside to warm up and charge the phone, which she accepted. Here, she rested in the bed next to the already sleeping defendant while waiting for the phone to charge. Although she remembered glimpses, the complainant admitted to the court that “due to intoxication, she only remembers fragments of the course of events, and is unsure of the order in which what happened.” She remembered rejecting the defendants’ sexual advances before she fell asleep. She then woke up from penetration, ran out, and called the police. DNA evidence was secured, and the defendant admitted that he must have had intercourse with the complainant, but that he could not remember it. He further expressed “strongly that it is completely against his nature to assault someone in the way he is accused of.”

The deciding minority acquitted the defendant in the criminal case, as they “could not rule out the possibility that the complainant could not be perceived as unconscious according to law or that she for other reasons was incapable of resisting the sexual intercourse with the defendant.” The minority proceeded to state in legal lingo that there is:

no necessary contradiction between believing that the complainant has accounted for the sequence of events the way she believes to remember that it happened, all the while we also find that there is uncertainty relative to what the appellate court can rely on as sufficiently proven about what state she was in when the sexual contact with the defendant was initiated.

They further questioned why the complainant did not leave the bed after the defendant's alleged first advances, before she fell asleep, “if he did behave as she explained.” The minority chose to give the defendant—who claimed not to remember anything about the assault—the benefit of doubt, while they questioned the memory, state, and behavior of the complainant, evoking victim blaming in the process. In stark contrast, the majority concluded “beyond any reasonable doubt that the defendant obtained intercourse with the complainant while she was in a state where she was unable to resist the act,” and further emphasized that “the defendant's claim that he cannot remember having had sexual intercourse with the complainant cannot be trusted [as] the accused only could have been moderately intoxicated at the time of the act.”

There is, as our case examples show, a tendency to interpret incapacity narrowly by requiring unconsciousness, and to give defendants leeway in terms of their responsibility to make sure their perceived sexual “partner” is indeed awake. Yet, sometimes the courts draw the boundary of un/consciousness in nuanced ways. LF-2020-47258 is a case in point. Here, the complainant had hosted a party, accommodated some of the guests, sent others home, and gone to bed in the early morning—to wake up a few hours later as the defendant was penetrating her anally. She got up abruptly, screaming. In the verdict the judges pointed out that it was credible that the complainant fell asleep shortly after having escorted the guests out in the early morning hours, given the amount of alcohol she had consumed, and after having hosted a 10 h long gathering. According to the verdict, the defendant explained that the complainant had been passive during the sexual encounter, which also suggested she was sleeping. Furthermore, the judges pointed out that she remembers the rape in flashes. While we have seen in other cases that such memories can be used as evidence of consciousness, and thus capacity to resist, this panel of judges explained:

The glimpses of memory from the incident only mean that [the complainant] had moments where she dozed or slept less heavily. She may have experienced moments of consciousness occasionally, but not enough to make it possible for her to resist [the defendants]'s action. It is not required that she slept, nor that she was physically unable to resist the action. It is sufficient that her mental capacity for resistance was gone.

In many 291b cases involving sleep, the complainants explain how once they became aware of their surroundings and the ongoing assault, they were shocked, could not move, or “froze.” In the last section of the analysis, we have included cases that concern “freeze” and/or “surprise” as “other reasons” for the complainants’ claimed or established incapacity to resist.

### Freeze and Surprise: “Other Reasons”

It is not rare for the complainant to describe a freeze response to sexual assault. Out of the 67 verdicts, 11 (16%) explicitly referenced a freeze reaction—all of which combined with either intoxication, sleep, or both. LB-2019-178835, one of a few stranger assault cases, presents the most explicit freeze reaction case in our material. Here, the complainant had been out drinking, and was waiting for a bus downtown Oslo at a much-trafficked bus stop, in the middle of the night. She was listening to music when a stranger sat down next to her and started to touch and lick her breast under her clothes. She froze and could not move; her body became stiff and paralyzed. According to the verdict, the defendant

touched her genitals under her panties, inserted one or more fingers into her vagina and licked her genitals. The actions were performed even though she turned away, pulled her shirt together over her chest, cried and said “stop” and “help me,” or something to that effect. But she was unable to resist the actions because she was under the influence of alcohol and/or due to fear and/or due to her mental state.

After a while, she managed to get eye contact with witnesses nearby who helped her and testified to that in the court case. This is the only case where the court summoned an expert to explain a freeze reaction to the court. A chief physician from the rape reception center testified that a freeze reaction is:

…an involuntary automatic or instinctive reaction to perceived danger that humans share with other animals. We know this from everyday speech in the terms “stiff from fear” or “terrified.” The freeze reaction is one of three main reaction patterns in perceived danger: the other two are flight or fight. The person does not think about—and does not consciously choose which reaction pattern is exercised, it happens automatically. The freeze reaction is triggered by strong fear, physical contact with an attacker and the experience of being «trapped», either by being physically held, but also, for humans, by the experience of a threatening and unsolvable overall situation (often unknown surroundings, threatening behavior). It can be both a complete paralysis, or degrees of perceived immobilization.

The case illustrates thus how a court can counter a typical rape myth (i.e., that a victim of a real rape screams, fights, or flights) by the use of expert evidence. Interestingly, while the court acknowledged that the defendant had been drinking, they explicitly downplayed the relevance of intoxication, emphasizing the freeze reaction in isolation:

…A recalculation indicates that she had an alcohol concentration at 1.5–1.7 per mille. However, the Appellate court is convinced that the victim, due to a strong sense of fear, had a freeze reaction which made her unable to move significantly or to raise her voice.

In other cases, it is the freeze reaction *in combination with* sleep and/or intoxication that produce incapacity. LF-2020-98571, a dissent-based decision, is a case in point. Here, the complainant “was in a state where she felt distant, (…) in and out of a *sleep-like condition*” (emphasis added) because of intoxication and fatigue. According to the majority,

the accused took off [the complainant's] trousers and panties [while she was lying on bed], so that she was only wearing a bra and a t-shirt. She registered that the accused performed oral sex on her, but she turned away and he stopped. She woke up again lying on her stomach as the defendant had penetrated her with his penis in her vagina. She then registered that he had a vaginal intercourse with her for a while before he left the room for a short while. When he returned, he had anal intercourse with her, after lubricating his penis with “lubricant.” The sexual intercourse ended when the accused ejaculated. He then pulled on her panties again.

The majority described how the complainant registered what the accused did to her, but was unable to react, and concluded as follows:

The majority finds that the complainant, after she woke up when the defendant was penetrating her, was aware of what was happening to her. Still, she could not resist the sexual intercourse that was going on, she just lay still, pretended to sleep, and let it happen. She felt then and there that she had no other choice and said in court that she was also scared and felt “paralyzed” in the situation.

By calling the complainant's state a “sleeplike condition,” the majority established an association to “sleep” and accordingly “unconsciousness,” while they at the same time downplayed the degree of consciousness that the minority argued made her capable of resisting the act. The minority, however, seemed to argue that even a minimum of consciousness makes a victim capable of resisting unwanted sexual acts. The dissenting minority concluded that it had not been proven “beyond any reasonable doubt that the victim was unconscious or for other reasons in such a physical or mental state that she was not able to resist the sexual intercourse.” In their deliberation, they pointed out that she explained how she pretended to be sleeping, that she chose to stay passive and to not attempt to resist the sexual intercourse verbally or physically, beyond turning away as the defendant performed oral sex on her. Additionally, the minority emphasized that they “cannot establish with sufficient certainty that the accused understood or was aware that the sexual intercourse *took place* because the victim was unable to resist it.” By such passive voice, the minority referred to the sexual intercourse not as an act of the defendant, but as “a thing that happened,” decidedly leaving it to the victim to resist sexual advances, rather than bestow on the defendant a responsibility to make sure that such advances are wanted.

A few 291b cases involve “other reasons” as separate and independent of sleep, intoxication, or freeze—including assault by strangers and surprise, abuse of position, and various physical conditions that make assault possible, and limit the complainants’ ability to physically resist (e.g., LB-2019-186741). For example, LB-2019-186741 concerns a defendant who was found guilty of following and attacking various women on their way home from public transportation, pushing his fingers up their vaginas. The case demonstrates how context is deemed relevant to incapacity considerations and emphasizes the ambush and surprise element of the assaults. This was also the case in LE-2019-191089. Here, the defendant, a gynecologist, was convicted of raping several patients when they were lying in his examination chair for a gynecological examination. The argument in this case was that the chair put their bodies in a position that impaired their physical ability to resist the sexual acts. The case was tried in combination with Section 295a: the defendant was found to have obtained sexual activity by abusing his position and a relationship of trust with his patients.

## Discussion

In 291b cases, what makes legal sense and qualifies for the threshold of “incapable of resisting” is left to the court actors’ interpretation in the free evaluation of evidence. In the presentation of findings above, we address the basis on which judges render incapacitated rape im/probable, seeking to understand practices of inference and sensemaking from recognition of their deliberations as a process of legal veridiction. We provide examples of how the line of in/capacity is drawn in relation to the four main categories for incapacity to resist that the appellate courts address: intoxication, sleep, “freeze,” and surprise. The analysis demonstrates how some courts draw the boundaries of rape in ways that seem to add rape myths to the legal process: about how to sleep as a credible victim: how deep and how fast or slow, how to blackout properly (consistently!), how to object sufficiently, and how to be reliably incapable of resisting. While courts practice law pertaining to 291b requirements differently, we are concerned that the paragraph seems often to be interpreted as less about the integrity and autonomy of the victim than it is about shielding many defendants’ inability to care, understand, or be interested in the complainants’ will, presence, or desires. Combined, our findings further point to three interrelated tendencies in appellate courts’ construction of incapacity for the purpose of concluding cases that we will elaborate on below: (a) unconsciousness is the dominating threshold for incapacity, (b) victim responsibilization and a presumption of innocence beyond theoretical doubt, and (c) forensics matter less than storied accounts of events in credibility assessments.

### Unconsciousness as Threshold for Incapacity and Resistance

While the legal text and its preparatory work open “for other reasons” than unconsciousness to qualify a victim of sexual assault to be “incapable of resisting,” we find that appellate courts often seek to determine whether “other reasons”—such as intoxication—is, in fact, unconsciousness as well. While Jacobsen (2019) argues that the formulation “*or* for other reasons” makes it unnecessary to draw sharp boundaries around un/consciousness and opens for other conditions to potentially constitute a state of incapacity, the courts tend to make “other reasons” a superfluous qualification. For “other reasons” to qualify the sexual “encounter” in question as rape for reasons of incapacity, Norwegian appellate courts discuss whether these other reasons are a state of unconsciousness. In this endeavor, the appellate courts equate unconsciousness with incapacity on the one hand while degrees of consciousness are associated with capacity to resist on the other. To establish whether the complainant was unconscious *enough* to be unable to resist the sexual advances of the defendant, the written deliberations focus on issues to do with bodily re/actions (see LB-2019-50934 above), memories of the alleged assault (see LB-2020-41177 above), and whether she spoke at the time (see LA-2019-173370 above). When courts make unconsciousness the threshold for the application of 291b rather than one of several sufficient factors that can dwindle victims’ capacity to resist and make an act rape, deliberations become ignorant of, e.g., power asymmetries at play, fears and survival instincts evoked by assaults, or the impact of intoxication on the complainants’ ability to resist, object, or perceive a situation for what it is—which does not start at medical unconsciousness. Where the court questions whether the complainant was unconscious, it leads to victim responsibilization.

### Victim Responsibilization and the Benefit of Theoretical Doubt

Victim responsibilization is evident in many 291b cases. For instance, one verdict suggests that the complainant could and should verbally resist with explicit language for the defendant to be able to understand that she did not want to have sexual intercourse with him (LA-2019-173370). Another bench questioned why the complainant in a case fell asleep and did not simply leave after she had rejected the defendant's sexual advances the first time (LG-2020-50105). In a third verdict illustrating victim responsibilization, a minority dissenting opinion pointed out that to stay passive during an assault is a choice (LF-2020-98571).

Furthermore, when sleep as unconsciousness becomes the normative principle that guides the legal gaze in the decision-making process, the court can question whether the complainant was really sleeping so deep, so fast, as to qualify for unconsciousness, rather whether their condition reduced their capacity to resist (LB-2018-119700). Sometimes this leads to acquittals, other times to victim-blaming in dissenting opinions. In our reading of these deliberations, we keep coming back to the term “resist.” It is a problematic and ill-defined qualifier that in several cases shifts the courts’ focus on responsibility from the defendant to the complainant, in that verdicts question whether the latter in a clear enough manner communicated that they did not want to have sex. There are concerning decisions and deliberations in our data material that suggest that a man (or woman) can assume that others want to have sex with them unless they are medically unconscious—and need only stop if the victim physically and manifestly resists the attempt—or at least attempt to do so. Otherwise, they are not expected to understand that they did not want to engage in sexual activity (see, e.g., LH-2019-155441, LA-2019-173370, LG-2020-50105, and LB-2018-119700). The analysis also points out how courts navigate between trusting the complainants’ accounts—i.e., considering complainants to be trustworthy—and finding their testimony to be unreliable because of alcohol consumption (e.g., LB-2020-41177). These cases illustrate how complainants’ memories of assault add up to a catch-22 situation in court deliberations pertaining to intoxication and sleep: if they remember too well, they were hardly intoxicated or unconscious enough for incapacity to be relevant; if they remember too little, their version of events cannot be trusted.

Overall, the deliberations in 291b cases tend to emphasize the condition of the victim more than the exploitative acts of the defendant. This follows from the phrasing of and defining criteria for 291b cases and is not surprising, as Jacobsen (2019) also has pointed out. The emphasis on the victim is common in rape cases in general, beyond 291b cases specifically, as rape victims increasingly become entangled in medical–legal networks that assess the victim's body as a crime scene from which forensic and psychological evidence need to be collected for a case to be raised (Laugerud, 2020b). Yet, the analysis clearly shows how this attention to observable and objective characteristics that signify the state of the complainant is not complemented by a corresponding investigation of the defendants’ interest in an effort to make sure that the complainant wanted and welcomed the sexual contact. In our view, this overall emphasis on traits of the complainant as a means through which the court assesses the culpability of the defendant is problematic—especially as we see several acquittals based on what we understand as theoretical (in contrast to reasonable) doubt. Verdicts often point out that the defendants’ account seems adapted to the situation, claims not to remember, or remembers that they crossed some boundaries, while describing the complainants’ account as credible and consistent. Verdicts can still end with acquittals concluding they cannot rule out the account of the defendant, including variants of the claim that he could not know if she was awake/conscious/participating, whether for darkness, bodily reactions, or the lack of outright physical, or loud and clear verbal, resistance.

### Forensic Truths

How the courts understand incapacity shape the ways in which it can be proved. We find that forensic truths, pieced together retrospectively by scrutinizing traces of matter such as blood samples to establish the degree of intoxication, matter less than storied accounts of witnesses’ observations and experiences (Valverde, 2015). That is, legal practice in 291b cases is less concerned with mathematical calculations of alcohol percentages in urine/blood and average estimations of bodily (re)actions during reduced consciousness due to alcohol or sleep. Overall, forensic evidence is sparsely present in the judgments in these cases. In the judges’ deliberations, the issue to resolve tends to be whether the victim could have resisted. These deliberations concern the parties’ credibility more than forensic, factual levels of, e.g., intoxication. This lesser role granted to forensic evidence indicates that for 291b cases, legal practice, deliberations, and decisions are *storied*, more than “scienced,” countering concern that (Norwegian appellate) courts are shifting from “a narrative-oriented site of [legal] practice” to “one governed by a logic of the database” (van Oorschot & Schinkel, 2015, pp. 525–526; see also Helgheim, 2012). In the cases that do include an expert on toxicology, expert knowledge does not appear to be conclusive, but rather to corroborate other evidence, particularly witness testimonies.

## Concluding Remarks

We set out with this analysis to discuss the boundaries of rape that are constructed by the legal system according to Section 291b of the Norwegian penal code and the courts’ interpretation practices. The tendencies we describe are not true for all cases; thus, our problematization is not applicable to all court decisions in 291b cases. Yet, they point to common practices across courts and panels and thus to problematic consequences of the legal veridiction and interpretation process which follows from the vaguely defined “incapacity” requirement of §291b. As such, the analysis strengthens our concern about victims’ (and offenders’) equality before the law. The legal imaginary of what acts constitute rape depends on the judges’ interpretation of the state of the complainant, which in turn is graded along vague continuums of degrees of “consciousness” and “capacity to resist”—where the defining features of “resist,” too, are vaguely formulated. Our analysis shows that this formulation of law limits the legal imaginary of rape and demonstrates how it allows judges to interpret rape into something which the complainant is responsible for preventing unless s/he is unconscious or taken by credible surprise.

While we see how the analysis can be used as an argument in the ongoing consent debate, we caution against such interpretation. If anything, our analysis problematizes court deliberations where the victims’ characteristics and ways to resists and/or react to assault become the grounds on which the court defines the culpability of the defendant. In our view, this overall weighting of the state, acts, or inactions of the complainant replaces a focus that could and should lie primarily on the acts and inactions of the defendant: whether the defendant was aware of the state of the complainant and made sure that s/he was awake, or sought to seize sexual opportunities in the gray zone and take advantage of a complainant whatever the state they were in. Our analysis suggests that this is where the focus of the rape legislation debate should be to address the limited legal imaginary and unclear threshold for an act to constitute rape in 291b cases.

## Notes

1. The estimate is based on a mapping conducted for Houge's research project *From medical facts to legal evidence*, based on a collaborative effort with the police to identify rape cases (§§ 291, 293, and 294) completed at trial and appellate court levels in 2019.

2. The Norwegian legislation on sexual offenses is covered by Chapter 26 of the Norwegian Penal Code (2005). The chapter consists of 30 sections, the most important to the current analysis being Section 291 Sexual assault, but also Section 292 on minimum penalty, and Section 294 on grossly negligent sexual assault (The Penal Code, 2005): “Section 291 Sexual assault. A penalty of imprisonment for a term not exceeding 10 years shall be applied to any person who
(a) obtains sexual activity through violence or threatening conduct (b) engages in sexual activity with a person who is unconscious or for other reasons incapable of resisting the act (c) through violence or threatening conduct makes a person engage in sexual activity with another person, or perform acts corresponding to sexual activity on himself/herself Section 292 Minimum penalty for sexual assault involving intercourse, etc. The penalty shall be imprisonment for a term of between 3 and 15 years if the sexual assault as specified in Section 291 included (a) insertion of the penis into the vagina or anus (b) insertion of the penis into the aggrieved person's mouth (c) insertion of an object into the vagina or anus (d) if the offender brought about a state as specified **in Section 291** (b) in order to obtain sexual activity” “Section 294 Grossly negligent sexual assault. Grossly negligent sexual assault is punishable by imprisonment for a term not exceeding 6 years. If circumstances as specified in Section 293 exist, the penalty shall be imprisonment for a term not exceeding 10 years.” It should also be noted that the legislation on rape is currently under evaluation by the Norwegian National Criminal Law Council (Straffelovrådet, n.a.). For more on the development of rape legislation in Norway, past and present, see Jacobsen & Skilbrei (2020).

3. “Gray zone” as used herein refers to sexual encounters where agency, will, pressure, memories, desire, the sexual interaction itself, and hence also the applicable jurisdiction are somewhat blurred, or “muddy,” typically due to intoxication (see, e.g., Cahill, 2016; Demant & Heinskou, 2011). The “gray zone” refers, thus, to sexual encounters in the ambiguous space in-between the binary constructed by clearly defined consensual sex on the one hand, and sexual violence on the other (see Hansen, 2023).

## Appendix: referenced judgments

**Table:**

LB-2020-41177	LB-2019-96629	LG-2018-190076	LB-2019-50934
LA-2019-173370	LH-2019-155441	LB-2018-169937	LH- 2019-136465
LB-2018-98851	LB-2019-17336	LG-2019-75089	LH-2019-119406
LG-2020-50105	LB-2018-125155	LB-2018-119700	LB-2019-171089
LF-2020-47258	LB-2019-178835	LF-2020-98571	LB-2019-186741
LE-2019-191089	HR-2003-368-A-Rt- 2003-687	HR-1993-115-A-Rt- 1993-963	
